# Dynamic Contrast-enhanced MRI Demonstrates Pulmonary Microvascular Abnormalities Months After SARS-CoV-2 Infection

**DOI:** 10.1164/rccm.202210-1884LE

**Published:** 2023-04-24

**Authors:** Iris Y. Zhou, Molly Mascia, George A. Alba, Michael Magaletta, Leo C. Ginns, Peter Caravan, Sydney B. Montesi

**Affiliations:** ^1^ Harvard Medical School, Boston, Massachusetts; and; ^2^ Athinoula A. Martinos Center for Biomedical Imaging, Department of Radiology,; ^3^ Institute for Innovation in Imaging, and; ^4^ Division of Pulmonary and Critical Care Medicine, Massachusetts General Hospital, Boston, Massachusetts

*To the Editor*:

Severe acute respiratory syndrome coronavirus 2 (SARS-CoV-2) causes vascular endothelial abnormalities (1). Pulmonary vascular dysfunction is a key component of severe SARS-CoV-2 infection (2). The extent of endothelial dysfunction in nonsevere infection and its sequelae are not fully understood. We and others recently showed that dynamic contrast-enhanced magnetic resonance imaging (DCE-MRI) could detect microvascular differences in patients with idiopathic pulmonary fibrosis (IPF) compared with healthy volunteers (3, 4). DCE-MRI involves continuous dynamic imaging before, during, and after injection of gadolinium-based contrast. The resultant signal intensity versus time curve provides information regarding microvascular perfusion and extravascular extracellular space (5). We hypothesized that DCE-MRI could detect microvascular changes in individuals with prior symptomatic SARS-CoV-2 infection.

Some study results have been reported in abstract format (6).

This study was approved by the Massachusetts General Brigham Institutional Review Board. All participants provided written informed consent. Individuals with prior coronavirus disease (COVID-19) who had a positive SARS-CoV-2 PCR test within the previous 3–12 months were recruited through the Massachusetts General Hospital Coronavirus Recovery Clinic. Healthy volunteers without known lung disease were recruited through a different protocol. Participants were excluded for respiratory illness within the past 6 weeks, cigarette smoking within the past 6 months, gadolinium allergy, or contraindications to MRI.

DCE-MRI was performed using a 3T MRI scanner (Siemens Healthineers) using a radial sampling sequence: flip angle, 15°; echo time, 1.0 ms; repetition time, 3.4 ms; field of view, 410 × 410 mm^2^; voxel size, 2.1 × 2.1 × 2.5 mm^3^; and 72 slices. Serial volumetric pulmonary images were acquired during free breathing and reconstructed to a temporal resolution of 2.75 seconds (7–10). Imaging acquisition started 60 seconds before injection of 0.05 mmol/kg gadoterate meglumine (Guerbet) at a rate of 4 ml/s and continued for 360 seconds. Signal intensity versus time curve was extracted for the entire lungs and for a region of interest (ROI) from a posterior coronal plane which included the lungs but excluded large vessels. We measured the magnitude of peak enhancement, the rate of contrast arrival (k_washin_), time to peak enhancement, the full width at half maximum for the peak (FWHM) as a surrogate of contrast transit time, and the rate of contrast washout (k_washout_), as defined previously (3). Prior COVID-19 participants completed the modified Medical Research Council (mMRC) dyspnea scale. Hospitalization status, pulmonary function testing (PFT), and chest computed tomography (CT) results were recorded from the medical record. Differences between groups were assessed using the Wilcoxon rank-sum test or Fisher’s exact test, with *P* < 0.05 considered statistically significant. Spearman’s rank correlation coefficient was used to assess the relationship between time from positive SARS-CoV-2 PCR test and MRI measurements.

Ten participants with prior COVID-19 (median age, 57 years; 50% male) and 10 healthy volunteers (median age, 43 years; 40% male) underwent DCE-MRI (Table 1). For participants with prior COVID-19, the average time from positive SARS-CoV-2 PCR test to DCE-MRI was 7.8 months. Positive SARS-CoV-2 PCR tests occurred between September 2020 and September 2021. Three participants required hospitalization for COVID-19, two of whom required supplemental oxygen (maximum flow rate, 4 L/min). Median mMRC score on the day of DCE-MRI was 1 (range = 0–2). All underwent clinically obtained spirometry, lung volumes, and Dl_CO_, and 5 had chest CT imaging performed after COVID-19 and before DCE-MRI. Two participants had mild restriction, with 1 having a mild diffusion impairment. One participant had borderline mild obstruction. Remaining PFT results were normal. Of the participants that had chest CT imaging, 3 had no parenchymal abnormalities, and 2 had minimal residual parenchymal abnormalities.

**Table 1.** Descriptive Statistics by Participants with Prior Coronavirus Disease versus Healthy Volunteer Groups

Variable	Participants with Prior COVID-19 (*n* = 10)	Healthy Volunteers (*n* = 10)	*P* Value
Age, median (range)	57 (37–76)	43 (27–74)	0.42*
Male, frequency (%)	5 (50)	4 (40)	>0.99^†^
mMRC, median (range)	1 (0–2)^‡^	—	—
Hospitalization for COVID-19, frequency (%)	3 (30)	—	—
Mechanical ventilation, frequency (%)	0 (0)	—	—
Noninvasive ventilation, frequency (%)	0 (0)	—	—
Supplemental oxygen, frequency (%)	2 (20)	—	—
FEV_1_,% predicted, mean ± SD	96.5 ± 15.2	—	—
FVC, % predicted, mean ± SD	98.6 ± 14.5	—	—
FEV_1_/FVC, mean ± SD	76.9 ± 4.6	—	—
Dl_CO_, % predicted, mean ± SD	99.4 ± 15.0	—	—
TLC, % predicted, mean ± SD	86.7 ± 14.2	—	—
Peak enhancement, %, median (range)			
Posterior coronal	210 (90–270)	250 (200–410)	0.06*
Whole lung	170 (80–250)	200 (160–340)	0.12*
TTP, min, median (range)			
Posterior coronal	0.12 (0.10–0.17)	0.11 (0.09–0.12)	0.25*
Whole lung	0.12 (0.10–0.16)	0.12 (0.11–0.13)	0.67*
k_washin_, %/min, median (range)			
Posterior coronal	1,900 (770–2,800)	2,400 (1,900–3,400)	0.04*
Whole lung	1,500 (770–2,200)	1,800 (1,500–2,600)	0.09*
FWHM, min, median (range)			
Posterior coronal	0.17 (0.11–0.38)	0.14 (0.11–0.15)	0.02*
Whole lung	0.17 (0.12–0.28)	0.15 (0.12–0.18)	0.44*
k_washout_, %/min, median (range)			
Posterior coronal	−5.60 (−10.50 to −2.10)	−6.10 (−13.30 to −3.40)	0.53*
Whole lung	−4.10 (−6.70 to −0.50)	−4.60 (−8.20 to −1.20)	0.58*

*Definition of abbreviations*: COVID-19 = coronavirus disease; FWHM = full width at half maximum; k_washin_ = rate of contrast arrival; k_washout_ = rate of contrast washout; mMRC = modified Medical Research Council dyspnea scale; TTP = time to peak enhancement.

Data are reported as mean ± SD, frequency (percentage), or median (range).

* Wilcoxon rank-sum test.

^†^ Fisher’s exact test.

^‡^ mMRC results performed at the time of MRI and were only available for 9 participants.

**Figure 1.**
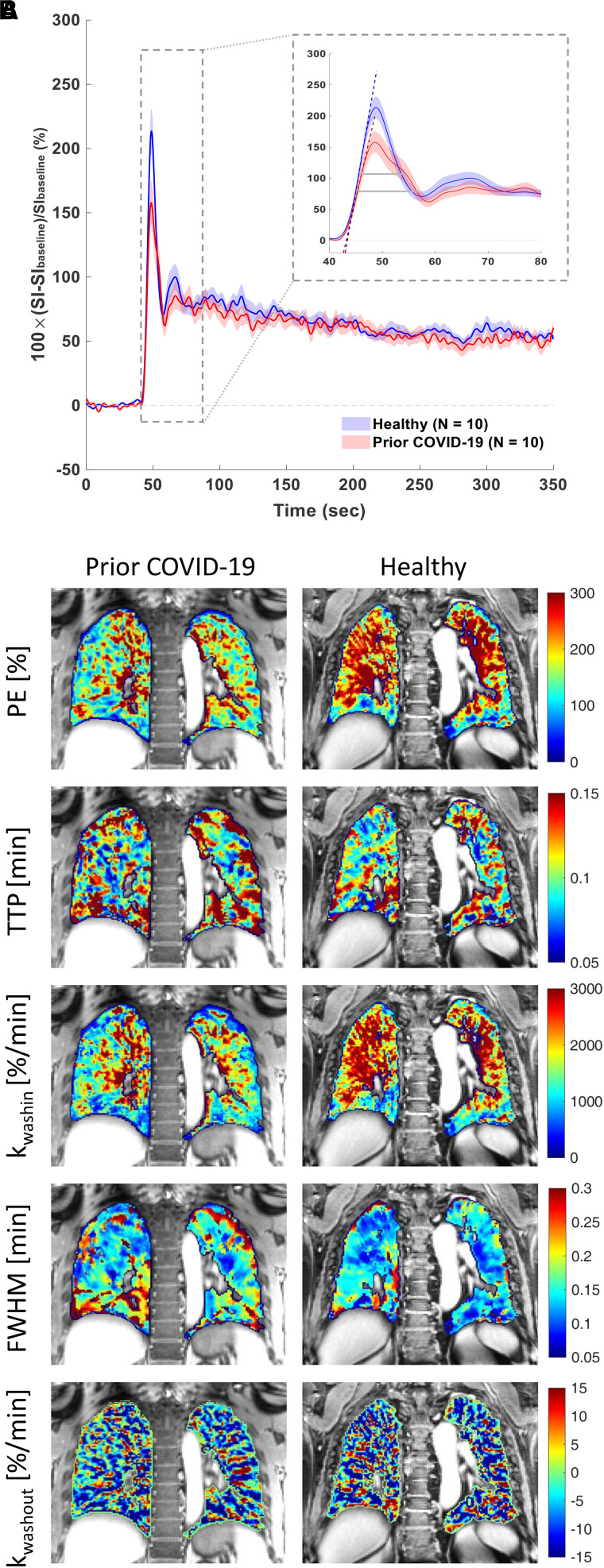
Dynamic contrast-enhanced magnetic resonance imaging of prior coronavirus disease participants versus healthy volunteers. (*A*) Magnetic resonance imaging signal intensity (SI) versus time curves of the lung parenchyma from healthy volunteers (*n* = 10) and participants with prior coronavirus disease (COVID-19) (*n* = 10) computed as percentage of SI change relative to the lung signal before gadolinium administration. The group-averaged dynamic curves from posterior coronal regions of interest are shown. Shaded area indicates mean ± 1 SEM. The inset shows the zoom-in of the first-pass peaks, with dashed lines indicating the upslopes and gray horizontal bars indicating the full width at half maximum (FWHM) of the first-pass peaks. (*B*) Representative parametric maps of peak enhancement, TTP, k_washin_, FWHM, and k_washout_ from a posterior coronal region of interest of a participant who had prior COVID-19 and who did not require hospitalization and a healthy volunteer. k_washin_ = rate of contrast washin; k_washout_ = late-phase washout slope between 60 seconds postinjection and the last acquisition; PE = peak enhancement; TTP = time to peak enhancement.

Group averaged signal versus time curves obtained from a posterior coronal ROI are shown in Figure 1A. Compared with healthy volunteers, participants with prior COVID-19 had signal versus time curves that were consistent with reduced pulmonary microvascular perfusion: The rate of contrast arrival to tissue (k_washin_) was significantly slower (1,900%/min vs. 2,400%/min), the peak itself was significantly broader (FWHM, 0.17 min vs. 0.14 min), and there was a trend toward a lower magnitude of peak enhancement (210% vs. 250%) (Table 1). Unlike in IPF participants, for whom the rate of contrast washout was significantly slower than for healthy volunteers (3), in this study, participants with prior COVID-19 showed no differences in k_washout_ between groups (−5.6%/min vs. −6.10%/min), and this suggests that there is no extravascular extracellular volume expansion occurring in the participants with prior COVID-19. There was a trend toward a slower k_washin_ in the analysis of the entire lungs (1,500%/min vs. 1,800%/min), although no microvascular parameters reached significance, indicating that the effect might be posterior predominant. Parametric maps of a posterior coronal ROI for a participant with prior COVID-19 demonstrate that measurements of microvascular perfusion are diffusely reduced throughout the lungs compared with those for a healthy volunteer (Figure 1B). There was a positive correlation between the time between positive SARS CoV-2 PCR testing and MRI and peak enhancement from the posterior lung (Spearman *r* = 0.66, *P* = 0.04) but not with k_washin_ or FWHM.

Our study has several important findings. First, in a small group of participants with remote and predominantly nonsevere COVID-19, pulmonary microvascular perfusion was reduced compared with age-similar healthy volunteers. Notably, 90% of the participants with prior COVID-19 had normal Dl_CO_ values, suggesting that DCE-MRI is more sensitive to detecting microvascular abnormalities. Second, rates of contrast washout were similar between groups, arguing against significant residual tissue fibrosis or edema.

There is growing interest in using functional lung imaging to assess postacute sequelae of SARS-CoV-2 infection. Yu and colleagues performed DCE-MRI in individuals with persistent dyspnea after COVID-19 and demonstrated a late bolus arrival of contrast compared with healthy volunteers (11). However, in that study, DCE-MRI was performed during a single breathhold (40 s), preventing extravascular extracellular space information from being obtained. Only perfusion parameters related to time to peak enhancement were analyzed. Grist and colleagues performed hyperpolarized ^129^Xe MRI in participants with breathlessness, despite normal to near-normal chest CT imaging at least 3 months after hospital discharge for COVID-19 and demonstrated reduced alveolar-capillary diffusion compared with healthy volunteers (12). Our results build on these findings and further demonstrate the sensitivity of DCE-MRI for detecting pulmonary microvascular pathology.

We are unable to draw associations with the DCE-MRI–derived measurements and CT findings, PFT abnormalities, or persistent dyspnea given our sample size. Although our results demonstrate the sensitivity of DCE-MRI for assessing microvascular differences, our small sample size may have prevented us from detecting other differences or correlations. For example, the variability in k_washout_ may mask possible changes in extracellular volume in a subset of subjects. Similarly, we observed a positive correlation with peak enhancement versus time from positive PCR, but this was not observed with k_washin_ or FWHM. Our results included participants who had COVID-19 before the emergence of the omicron variant. It is plausible that different strains of SARS-CoV-2 have different predilections for causing pulmonary vascular dysfunction. We cannot confirm the absence of prior SARS-CoV-2 infection in the healthy volunteers. We acknowledge the potential for selection bias in the recruitment of participants with prior SARS-CoV-2 infection as the degree of symptomatology could have influenced referral to our Coronavirus Recovery Clinic.

Our results support microvascular perfusion abnormalities months after COVID-19. The driver of these findings, such as residual microvascular thrombosis or sequelae of vascular remodeling, remains to be determined (13, 14). Understanding the pathophysiologic underpinnings of our findings may have therapeutic relevance for acute SARS-CoV-2 infection and postacute sequelae of SARS-CoV-2 infection.
